# Adaptive E-Nose: Integrating New Gas Sensors for Emerging Applications

**DOI:** 10.3390/s26134049

**Published:** 2026-06-25

**Authors:** Namkha Gyeltshen, Adrian Garrido Sanchis, Nishant Jagannath, Savindu Radaliyagoda, Sonam Tobgay, Md Farhad Hossain, Kumudu Munasinghe

**Affiliations:** 1 School of IT and Systems, University of Canberra, Canberra, ACT 2617, Australia; nishant.jagannath@canberra.edu.au (N.J.); savindu.radaliyagoda@canberra.edu.au (S.R.); sonam.tobgay@canberra.edu.au (S.T.); mdfarhad.hossain@canberra.edu.au (M.F.H.); kumudu.munasinghe@canberra.edu.au (K.M.); 2 School of Science, University of New South Wales Canberra, Canberra, ACT 2610, Australia; adrian.garridosanchis@unsw.edu.au

**Keywords:** electronic noses, framework, gas-sensing systems, low-cost, sensor arrays, selectivity, VOCs

## Abstract

Conventional chemical analysis relies on costly laboratory instrumentation, while current e-nose systems are expensive for widespread deployment. New opportunities for low-cost, accessible e-nose applications are emerging for diverse fields due to the rapid evolution of inexpensive sensor technologies. We developed a framework that enables rapid integration of newly available low-cost gas sensors into functional e-nose systems, continuously evaluating them as they become commercially available. By characterizing their performance in multi-sensor arrays that mimic biological olfaction, the framework demonstrates effective odor discrimination in a low-cost e-nose system through coordinated behavior of a heterogeneous sensor array. Our testing approach includes sensor sensitivity, selectivity, and stability, which are to be combined with appropriate pattern recognition and AI algorithms in the future for effective chemical discrimination. This work provides a pathway for continuously updating e-nose technology with the latest available sensors in a cost-effective manner, thereby making advanced chemical sensing accessible for resource-limited settings and enabling large-scale deployment in real-world applications with future potential applications such as food quality monitoring, environmental sensing, smart agriculture, etc.

## 1. Introduction

All life forms sense odors or gather chemicals (e.g., volatile organic compounds (VOCs)) to collect vital information about their surroundings for survival. Terrestrial vertebrates have a nose with a dual role in olfaction (smell) and respiration (breathing) [1,2]; aquatic vertebrates have nostrils (nares) [3]; invertebrates have chemical sensors (chemoreceptors) in their bodies [4], and plants have specialized receptors at a cellular level [5]. Although other senses have been technologically replicated, such as cameras for sight and microphones for hearing, the sense of smell [6] has been difficult to replicate. This is because the biological olfactory system is so sophisticated, being made up of intricate turbinates and millions of receptors to create unique signal patterns for thousands of odorants in milliseconds.

This highly refined biological olfactory function has long driven scientists and engineers to innovate and design systems to emulate it, leading to the development of artificial olfaction systems known as electronic noses (e-noses): systems comprising a variety of non-specific sensors and advanced processors, mimicking nature’s remarkable sensitivity and pattern-recognition capabilities. These systems help us understand and solve problems in various fields of application, such as food spoilage detection and quality control, environmental monitoring, medical diagnostics through breath analysis, and the identification of hazardous substances, including explosives and chemical threats. Although not a physical organ for scent, this concept provides a heightened, often hyper-vigilant, sensitivity to materials deemed offensive, dangerous, or simply inappropriate for public safety. This type of “nose” does not sniff out pheromones but rather sniffs out data from metal oxide sensors, which is the result of a series of chemical reactions. For example, calorimetry sensors respond to thermal changes, whereas optical sensors react to specific light wavelengths. These signals can then be used to detect, identify, or measure target compounds, resulting in a safe environment through effective threat screening and filtering.

Since the conceptual development of a practical sensor array in the 1980s [7] and the subsequent definition of the term “electronic nose” in 1994 [8], the technology has led to the development of e-noses [9] for applications in various domains such as waste management [10], medical applications [11,12,13] and agriculture and forestry [14].

In recent years, innovative technology developments in electronic nose (e-nose) systems have emerged and aim to enhance odor classifications through advanced multivariate analysis and machine learning techniques. Traditional analytical techniques, which are time-consuming and expensive, requiring extensive sample preparation and trained technicians, have given room for the development of various e-noses, such as those based on single-wall carbon nanotubes (SWCNTs) in the wine industry [15], VOC pattern recognition and multivariate signal analysis in the medical industry [16], the detection of volatile organic gases through multivariate analysis in manufacturing industries [17], and machine learning methods for analysis of multidimensional signals in wastewater treatment [18,19].

However, despite innovations and advancements, current electronic nose technology still faces significant challenges, and there is always room for improvement in effectiveness in real-world applications.

(a) Low Specificity (Poor Selectivity) and Environmental Interferences
  The most fundamental challenge is the low specificity (sensors reacting to all compounds rather than only one chemical) of the sensors, especially for the metal oxide (MOS) type. These sensors are broadly cross-sensitive and make it difficult to distinguish between complex mixtures (like coffee aroma or polluted air) that share overlapping volatile organic compounds (VOCs) [20], as the highly ambiguous signal is difficult to deconstruct. This ambiguity is severely worsened by environmental factors, as sensor responses are highly susceptible to changes in ambient humidity and temperature [21], which can alter the sensor’s baseline and sensitivity, leading to inaccurate and highly unreliable readings in real-world conditions. Studies on Fe-doped SnO_2_ Acetone Sensors [22] showed how physisorption (physical adsorption) and chemisorption (chemical adsorption) control the gas-sensing process. Physisorption plays a major role at room and lower temperatures, maintaining stability, high sensitivity and strong selectivity toward acetone. As the operating temperature rises, chemisorption becomes more significant, boosting surface reactivity and sensitivity. However, this thermally activated condition can also raise the chance of cross-sensitivity to other gases, which might reduce the sensor’s selectivity.
(b) Sensor Drift
  The sensor is a device made up of active and passive materials to detect physical, chemical, or biological changes in its environment and convert them into output signals that are comprehensible. However, within real-world applications, sensor drift—a slow progressive change—occurs in the output signal without any corresponding changes in the actual quantity being measured. So sensor drift is arguably the most significant barrier to the long-term reliability of electronic nose technology [23]. This phenomenon is caused by physical and chemical degradation (aging) of the sensing material (e.g., the metal oxide layer) and “poisoning” from irreversible chemical binding. This drift means that the “fingerprint” pattern a sensor array produces for a specific odor today will be different from the pattern it produces weeks or months later, invalidating the trained machine learning model and forcing frequent, time-consuming recalibrations of the entire system. Sensor drift is limited by various factors such as extreme temperature fluctuations, constant mechanical stress, electrical noise and interference, sensor component aging, and sensor surface contamination and corrosion. Sensor drift can be subtle, but there might be an impactful issue that undermines the very purpose of measurement. So sensor-drift compensation [24,25,26] is necessary to ensure long-term reliability and accuracy. However, this aspect of sensors is not covered within this study; we currently focus on establishing a proof-of-concept and demonstrating the feasibility of the proposed heterogeneous sensor array framework across multiple applications.
(c) Bulkiness and High-Power Consumption
  While e-nose systems have evolved toward greater portability, their inefficient system design makes them unsuitable for flexible field deployment. Even though the core components, such as gas sensor arrays, transmission paths, microprocessors, and pattern recognition algorithms [9], are necessary for operation, the true portability and accessibility are compromised by their conventional integration. More importantly, despite their superior sensing capabilities, the widely used MOS sensors [27,28,29]—the Figaro Series, such as TGS2600 and TGS3870; Winsen Series, such as MQ-2, MQ-8, MQ-135, and Alphasense Series, such as MiCS-6814—have historically required integrated heaters that operate at 100–500 °C [30], consuming significant power that prevents long-term battery operation. Due to this design limitation, current e-nose systems can only be used in laboratory or stationary industrial environments, which restricts their use in dynamic field situations where compact, energy-efficient operation is crucial.

The shortfalls mentioned above in numerous electronic nose systems have motivated us to develop a novel approach to developing a framework to test and validate new sensors coming into the market; new flexible algorithms are made available, providing optimum portability for varied applications. Researchers have conducted studies on various substances, such as apple [31,32,33], mango ginger [34], etc., but only on the existing, established e-nose system. In this study, we have designed a novel system using low-cost gas sensors that will provide optimum analysis of odor signatures and a deep understanding of how they react to various aroma characteristics, capturing a unique gas signature for each substance. It can be used for diverse applications as required, ranging from the detection of explosives, the monitoring of food spoilage, water quality, and the environment, to the identification of hazardous substances.

## 2. Materials and Methods

### 2.1. Overview of Sensing Array

The array of sensors in an electronic nose system is its core component, acting as an “artificial olfactory receptor” for the device. These sensors are designed to mimic the non-specific yet comprehensive sensing ability, as that of the biological nose, in recognizing and classifying odors. Typically, an array contains different kinds of chemical sensors: homogeneous (same type, or slight variations) or heterogeneous (different types) as in Table 1.

Based on the applications, the sensors in the array collect electrical signals in a multidimensional response pattern, which can be analyzed by computer-system algorithms trained using a database of known odor “fingerprints.” The new odor pattern is compared with the patterns in the library and is then classified and identified as the closest match, effectively translating the unique fingerprint into a recognized smell (e.g., fruit, contaminant, etc.).

### 2.2. Experimental System Setup

**Figure 1:**
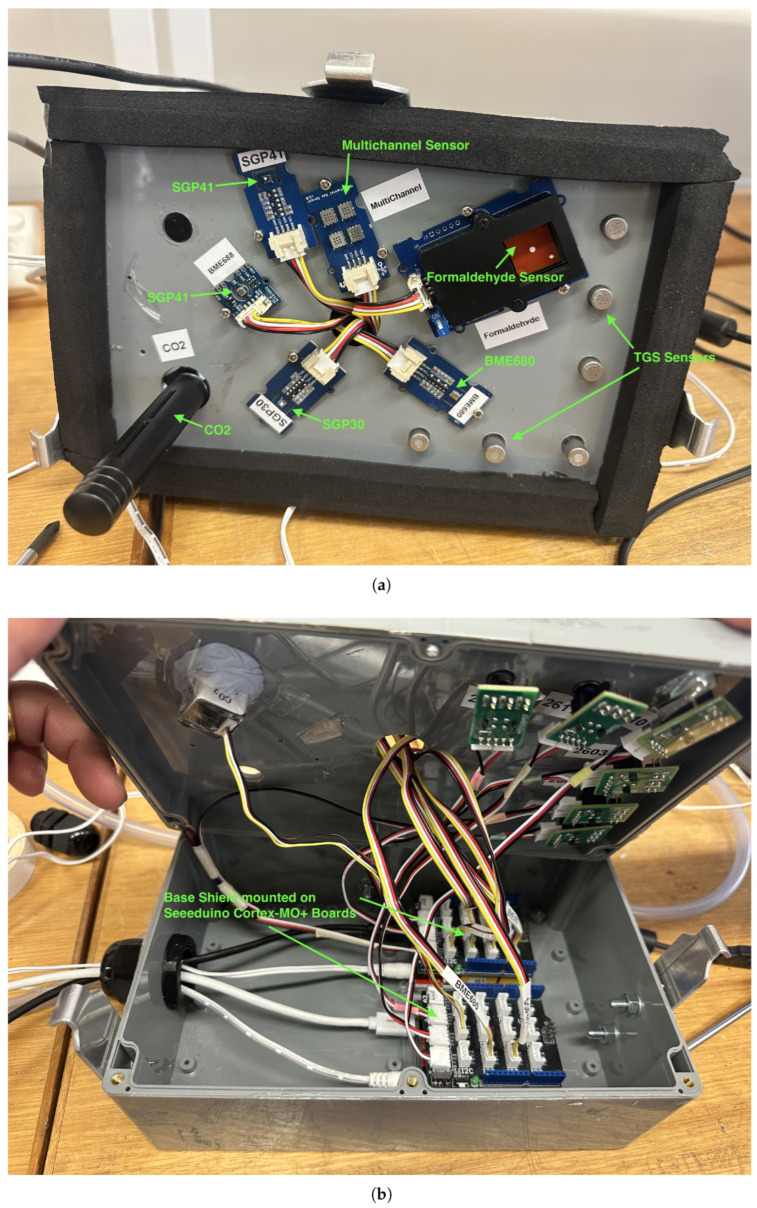

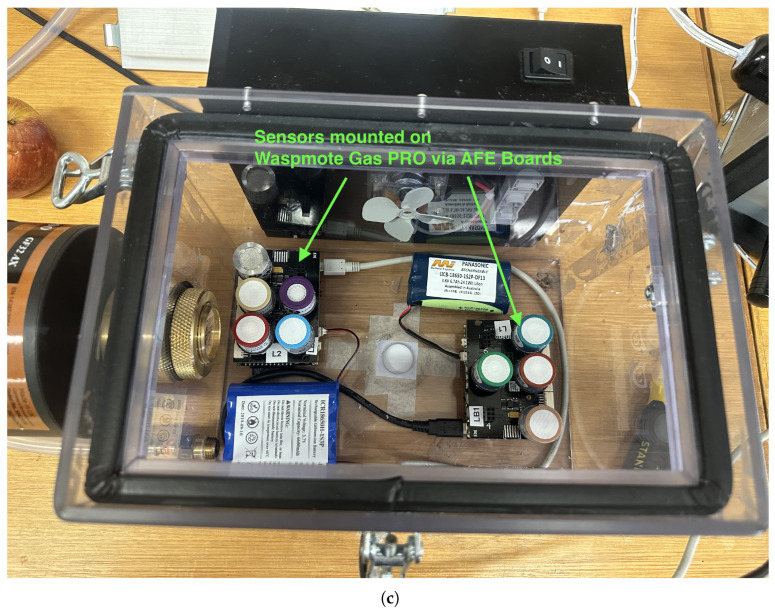
e-nose system setup. (**a**) Sensor array; (**b**) sensors connected to ARM Cortex-M0+ via the base shield; (**c**) electrochemical sensors mounted on a Waspmote Gases PRO Sensor Board via Analog Front End (AFE) boards.

In this experimental system setup (Figure 1), we have used an array of sensors that are exposed to various kinds of gases. The Metal Oxide Semiconductor (MOS) sensors, such as TGS2600 (Figaro Engineering Inc., Minoh, Japan), TGS2602 (Figaro Engineering Inc., Minoh, Japan), TGS2603 (Figaro Engineering Inc., Minoh, Japan), BME688 (Seeed Studio Technologies, Shenzhen, China), and SGP30 (Seeed Studio Technologies, Shenzhen, China), Multichannel Sensor (Seeed Studio Technologies, Shenzhen, China), and electrochemical sensor, such as SFA30 (Seeed Studio Technologies, Shenzhen, China), are grouped and connected in one board, as in Table A1, while other MOx sensors, such as TGS2610 (Figaro Engineering Inc., Minoh, Japan), TGS2611 (Figaro Engineering Inc., Minoh, Japan), TGS2612 (Figaro Engineering Inc., Minoh, Japan), BME680 (Seeed Studio Technologies, Shenzhen, China), SGP41(Seeed Studio Technologies, Shenzhen, China) and Non-Dispersive Infrared (NDIR) CO_2_ gas Sensors (Seeed Studio Technologies, Shenzhen, China) are placed together in another board, as in Table A2.

Additionally, to provide more diversity to the sensing process, eight calibrated Libelium sensors (Libelium Comunicaciones Distribuidas, Zaragoza, Aragon, Spain) (Table A3 and Table A4) mounted on two Waspmote Gases PRO Sensor Boards (Libelium Comunicaciones Distribuidas, Zaragoza, Spain), featuring the ATmega1281 microcontroller, have been integrated into the system. To each board, a set of batteries is attached to power the Real-Time Clock (RTC).

They are all housed in a 5.4 L inner capacity test chamber of size 235W × 180D × 210H (mm), fitted with a small mixing fan to circulate the inner air uniformly. The array of sensors that are connected to the Seeeduino boards (Seeed Studio Technologies, Shenzhen, China) has been installed in the upper chamber, and Libelium sensors mounted on the Waspmote Gases PRO sensor board are installed in the lower chamber. The chamber also has an air filter attached to maintain the internal environment optimally. An external 5 VDC power supply is supplied to all boards to stabilize the sensor readings.

### 2.3. Sensors and Specifications

The sensors are an integral part of the electronic nose system, and an array of them would constitute multiple gas sensors that respond to various gases. The different sensor technologies [35] capture a unique fingerprint produced by gases. Existing pattern recognition algorithms [36] can be used to identify and quantify the gases with more accuracy, despite the cross-sensitivity of the individual sensor. In this experimental setup, we have included the following sensors (in Table 2) and organized them into their respective development board groups.

Detailed specifications for every sensor mounted on the four acquisition boards—including sensing technology, target gases, measurement range, and cross-sensitivity—together with the individual sensor descriptions, are provided in Appendix A (Table A1, Table A2, Table A3 and Table A4).

### 2.4. Fruits and Their Scent

**Figure 2:**
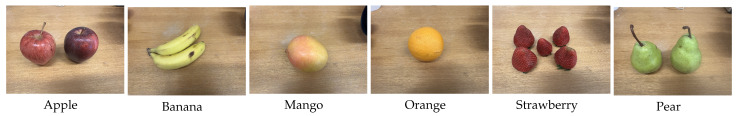
Fruit samples.

Fruit scent determines the quality of the fruit, providing the consumer with the best indicator of fruit flavor. The wide variety of volatile organic compounds (VOCs) released by fruits (in Table 3) is studied to identify, characterize, and grade different fruits [37]. The fruits usually produce chemical compounds that help in either ripening or contributing to aroma. The following fruits (Figure 2) have been tested for their contained compounds, as observed depending on their respective cultivar, harvesting, aging, etc.

### 2.5. Software System and Data Acquisition

Sensor data were acquired, stored, and visualized in real time by a modular software stack. Python (v3.14.6) acquisition scripts running on a central computer stream readings from the four sensor boards, save them to local CSV files, and upload them to a Firebase Realtime Database cloud backend, while a browser-based Plotly (v6.8.0) Dash dashboard provides live monitoring and experiment control.

#### 2.5.1. Architecture Overview

**Figure 3:**
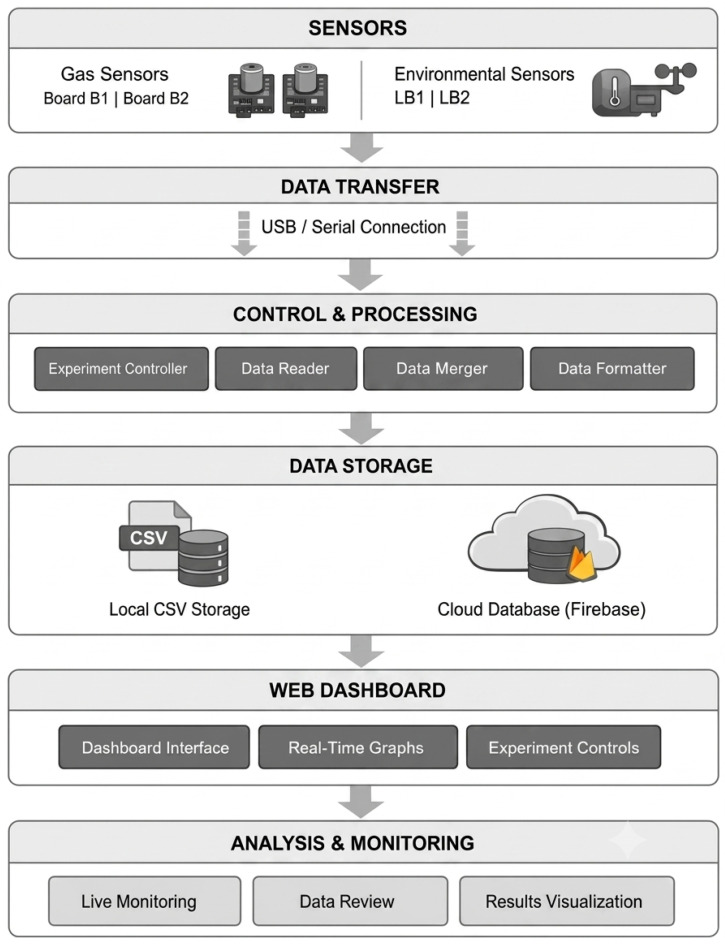
e-nose system architecture.

The e-nose software system architecture, as seen in Figure 3, has been developed to reliably collect, store, and visualize sensor data in real time during laboratory experiments. It connects four sensor nodes—two custom gas-sensing boards (B1 and B2) and two Libelium environmental boards (LB1 and LB2)—to a central acquisition computer that controls the experiment workflow.

Python scripts running on the acquisition computer handle serial communication with all sensor boards, process incoming data, save readings to local CSV files, and upload the same information to a cloud database. A web-based dashboard built using the Dash framework allows researchers to configure experiments and observe sensor responses live through a standard web browser.

This layered design allows the system to remain modular and easy to extend. For example, new sensors can be added without changing the overall architecture, and the dashboard can be accessed remotely without installing additional software.

#### 2.5.2. Data Acquisition Workflow

**Figure 4:**
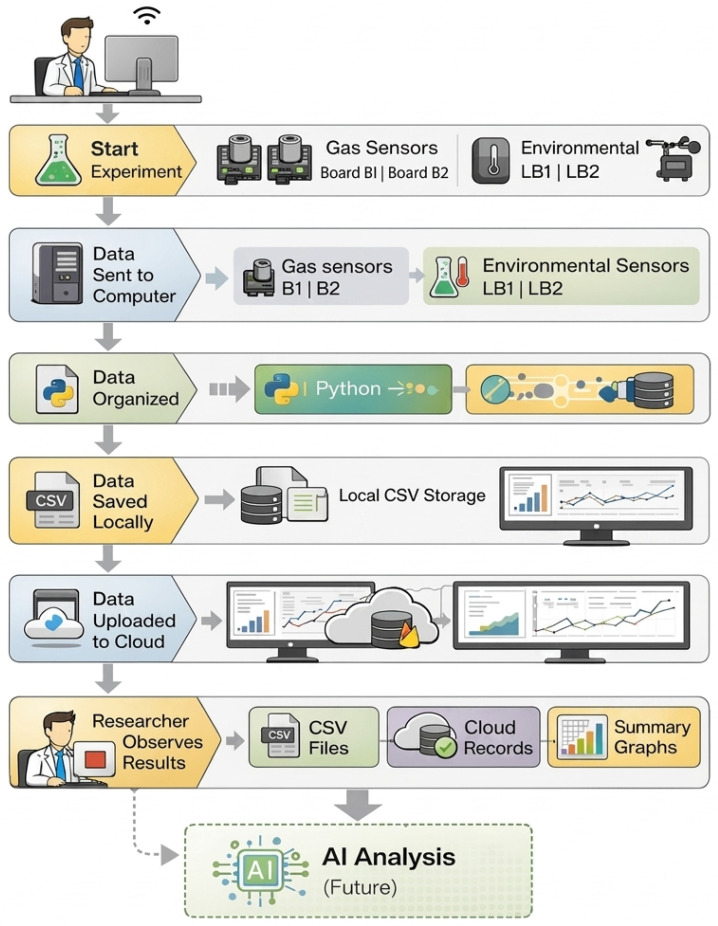
End-to-end workflow of the e-nose monitoring system.

The acquisition engine is written in Python and is responsible for all communication between the sensor hardware and the software system. A central script, write.py, controls the entire acquisition process. The sequence of data flow in the system is as shown in Figure 4. It reads user-defined experiment parameters from the dashboard, creates the appropriate directory structure, and starts the board-specific data listeners. Separate scripts handle different board types: one for the high-frequency gas sensors (B1 and B2) and another for the environmental sensors (LB1 and LB2). These scripts run in parallel threads so that data collection is not interrupted when one board responds more slowly than others. This modular approach ensures stable data collection even when the system is under heavy load or when individual boards behave unpredictably.

Further implementation details—the dashboard interface, the data collection and synchronization strategy, Firebase integration, and the real-time analytics—are provided in Appendix B.

### 2.6. Experimental Process

The experiment was conducted by initially fanning out any contaminants inside the room and the test chamber. After it is ensured that the setup is free of unwanted gases, the test chamber is closed without any samples inside it. The 220 VAC power supply is connected to the test chamber to run the mixing fan inside, and a 5 VDC supply to power up all the boards. The system is then kept running for 30 min to stabilize the sensor readings.

**Figure 5:**
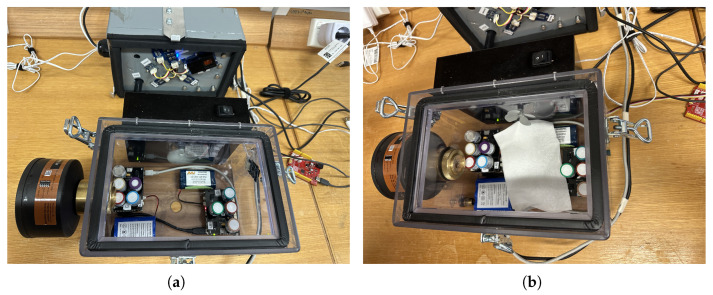

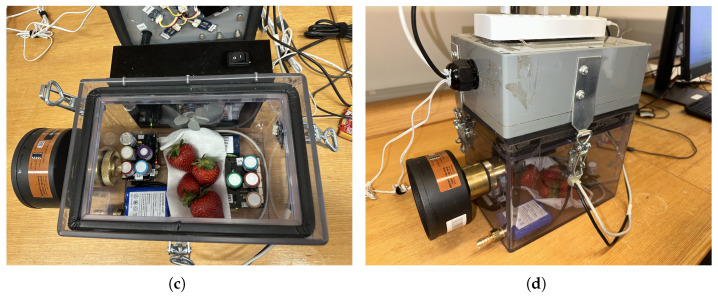
(**a**) Test chamber kept open. (**b**) The paper base is placed inside the test chamber. (**c**) Substance sample placed inside the test chamber. (**d**) Test chamber enclosed, with the sample inside it.

The baseline measurements are captured and recorded for 20 min. The test chamber is opened, and a piece of paper is placed inside as a base for the substance sample so that no aroma or scent is left on the floor of the chamber to avoid cross-contamination of odor. After each test is completed, it is replaced with a new paper to maintain the purity of the air inside the chamber. A substance sample is placed on the paper base, and the internal mixing fan evenly distributes accumulated gases. The process is kept running for 20 min, and data is captured and recorded both locally in a CSV file and uploaded in real time to the Firebase cloud. Once the data recording for a specific substance (e.g., fruit) is completed, the test chamber is opened to let out any gases accumulated inside the chamber. The test process was diligently repeated for all other substances. For the current set of experiments, we focused on the gases released by fruits, and no specific gas was injected. However, for future experiments, in which actual gases are to be introduced into the chamber, a hole has been provisioned at the bottom, as seen in Figure 1c. The sequence of the experimental process conducted in a laboratory setup is shown in Figure 5 below.

### 2.7. Tools for Statistical Analysis

All statistical analyses were conducted using IBM SPSS Statistics Version 30. Parametric statistical methods were applied depending on data distribution characteristics. Normality of the sensor response datasets was assessed using the Shapiro–Wilk test, which guided the selection of subsequent analyses. For normally distributed data, one-way analysis of variance (ANOVA) was performed, followed by Tukey–Kramer post hoc comparisons to identify statistically significant pairwise differences between fruit samples.

The recorded sensor responses represent time series measurements obtained during controlled exposure experiments, rather than independent biological replicates. Each fruit sample was measured over a defined exposure period, generating 52 temporal observations under identical conditions. These time-resolved data were used to characterize sensor response dynamics; however, they are not treated as statistically independent replicates in the strict experimental sense. Instead, the analysis focuses on identifying consistent and reproducible response patterns across substances, supported by both univariate (ANOVA) and multivariate methods.

In addition to univariate analysis, multivariate analysis was conducted using Principal Component Analysis (PCA) applied to standardized sensor-response features. PCA was used to reduce dimensionality, identify dominant variance patterns, and evaluate the overall structure of the dataset. This approach enabled visualization of clustering behavior and assessment of the ability of the e-nose system to discriminate between fruit types.

## 3. Results and Discussions

Existing studies have shown that ethylene is either released by fruits naturally or is used commercially to alleviate the ripening process [44,45]. As the ripening process increases, carbon dioxide [46] is released as a normal metabolic product, along with water vapor [47], which would raise humidity in a closed container. Additionally, many VOCs [48,49], such as esters, alcohols, aldehydes and terpenes, are released by fruits, which give the characteristic smell of each fruit. The VOCs are typically present at low concentrations compared with carbon dioxide and ethylene, but they are important for flavor and perceived freshness [47]. For this reason, many studies [32,34,38,39,43] have been conducted on VOCs released from fruits, and the output of these studies by other authors has helped us in better understanding the gases released by the fruits used in our experiment. So, in this study, out of the many gases detected, we have focused on the sensor’s responses to the VOC and CO_2_ equivalent, in accordance with our main objective of examining the selectivity and sensitivity of low-cost sensors for emerging applications.

### 3.1. Statistical Analysis

#### 3.1.1. Grove—Air Quality Sensor (BME688) Response to VOC and Carbon Dioxide (CO_2_) Equivalent Gas

**Figure 6:**
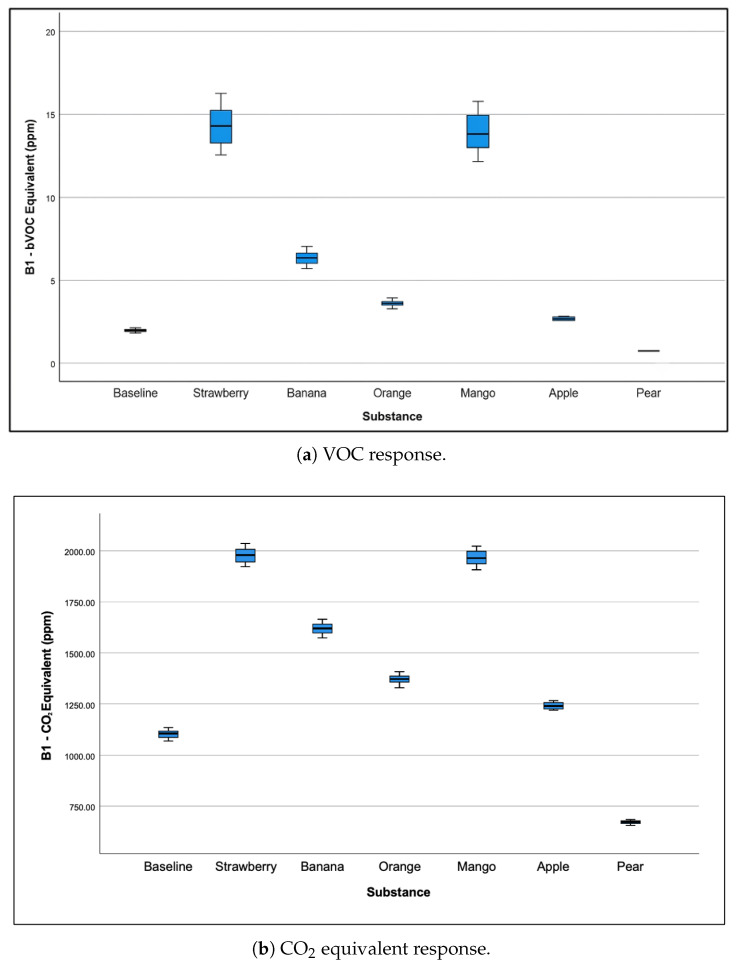

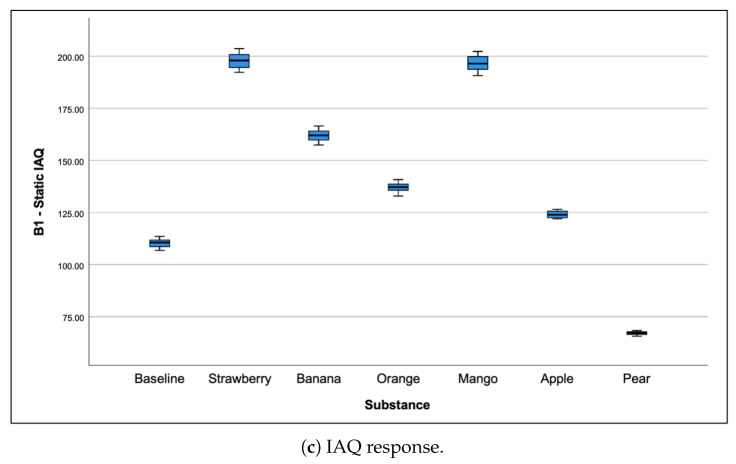
Grove—Air Quality Sensor (BME688) responses.

The boxplots in Figure 6a,b below show that the air quality sensor (BME688) clearly differentiates between the baseline background and all tested fruits using both VOC and CO_2_ equivalent outputs. Statistical analysis (one-way ANOVA, F = 4522, df = 6, *p* < 0.001; Dunnett T3, 95% CI) provides strong evidence that the VOC responses are significantly different across fruit types. The BME688 VOCs metal oxide-sensing element exhibits known cross-sensitivity to a wide range of volatile compounds released by fruits, enabling effective discrimination based on their vapor signatures.

A similar trend is evident in the CO_2_ equivalent channel (one-way ANOVA, F = 19,693, df = 6, *p* < 0.001; Dunnett T3, 95% CI). Despite not directly detecting CO_2_ from the fruits, the sensor interprets reducing VOCs as CO_2_ equivalent signals due to its chemical response mechanism. As a result, the CO_2_ equivalent output also successfully separates fruit samples from the baseline air.

Across both channels, all fruit types produce significantly higher responses than the baseline, with distinct median values and minimal overlap. Strawberry and mango generate the strongest responses, consistent with their high emission of volatile aromatic compounds. Banana and orange show intermediate responses, while apple and pear produce lower signals but remain clearly distinguishable from the baseline. Although fruits are not sources of CO_2_, the elevated CO_2_ equivalent readings reflect the sensor’s cross-sensitivity to reducing VOCs. This effect can be advantageously exploited for odor-based classification.

As illustrated in Figure 6c, the VOC equivalent and CO_2_ equivalent responses separate all fruit types with high statistical confidence (ANOVA: F = 19,693, df = 6, *p* < 0.001; Dunnett T3, 95% CI). Although BME688 does not directly detect CO_2_, its MOS-based gas-sensing mechanism interprets reducing VOCs as CO_2_ equivalent signals—a behavior that enhances pattern recognition capability for VOC-rich substances such as fruits.

Strawberry and mango samples consistently produce the strongest responses, reflecting their complex VOC emissions, while banana and orange show intermediate patterns. Apple and pear display lower signals but remain clearly differentiated from the baseline. The particularly low response from pear samples may relate to limited VOC release at the measured ripeness stage or a VOC profile that partially falls outside the sensor’s optimal sensitivity range.

#### 3.1.2. Grove—Air Quality Sensor (SGP30) Response to Carbon Dioxide (CO_2_) Equivalent Gas

**Figure 7:**
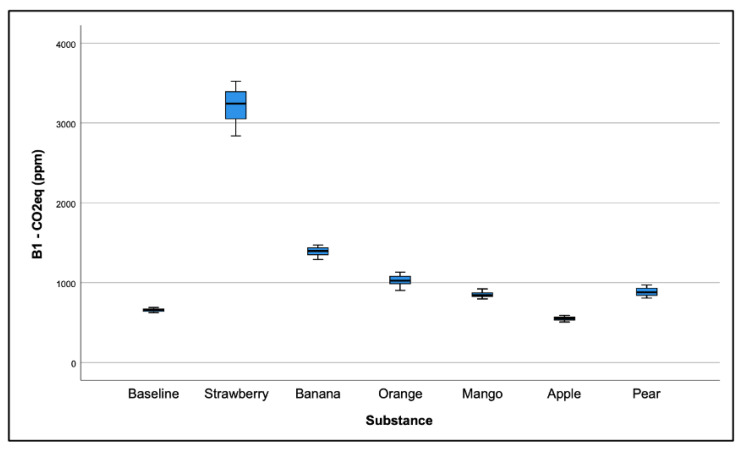
Grove—Air Quality Sensor (SGP30) responses.

**Figure 8:**
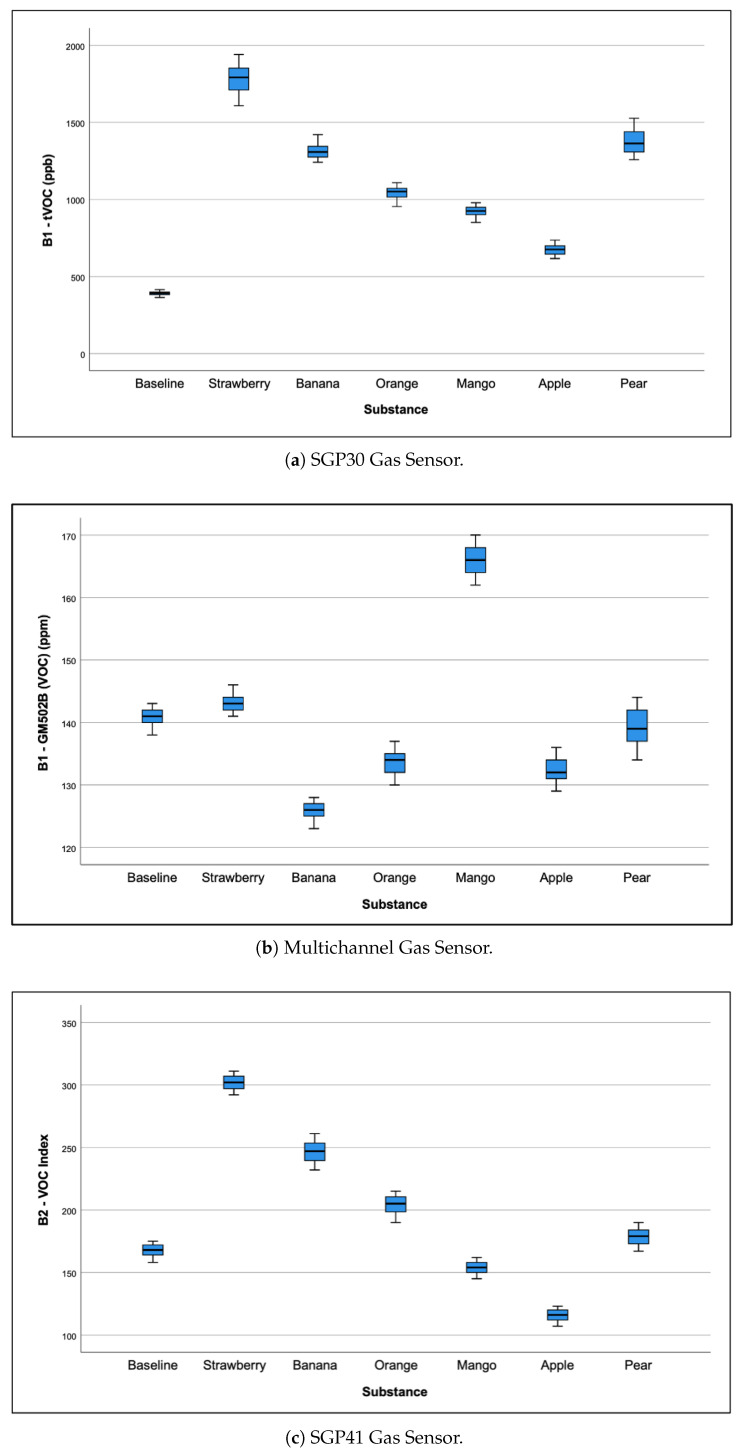
Comparison of VOC sensor responses.

Figure 7 shows the SGP30 sensor’s response to measuring CO_2_ equivalent in ppm across different fruit samples. The sensor has an exceptionally high response to strawberries, while the responses from other fruit samples are close to the baseline measurement. This is because SGP30 is a metal oxide (MOx) gas sensor that uses multiple sensing elements to detect volatile organic compounds (VOCs) and calculates CO_2_ equivalent values based on the total VOC concentration (Figure 8a) it detects, rather than actual CO_2_. Since strawberries are rich in distinct VOCs that give them a unique and abundant aromatic compound profile, the sensor shows high cross-sensitivity to those compounds, and it measures CO_2_ equivalent by detecting and quantifying the total VOC load, which is significantly higher in strawberries.

#### 3.1.3. Responses of Various Sensors to VOCs

Previous studies employing GC-MS technology have consistently shown that strawberries emit a complex mixture of volatile organic compounds (VOCs) [50], including esters, alcohols, aldehydes, terpenes, and lactones, with concentrations strongly influenced by cultivar, ripeness stage, and post-harvest handling conditions. These VOCs form the strawberry’s characteristic aroma signature and provide a useful benchmark for assessing sensor performance. To determine which of our sensing technologies are most effective in capturing these signatures, strawberries were placed inside a controlled test chamber and evaluated using multiple gas sensor platforms.

Figure 6a and Figure 8a demonstrate that both the BME688 and SGP30 sensors detect markedly elevated VOC levels across all tested fruits, with especially pronounced responses for strawberries. Statistical analysis for Figure 8a confirms significant differences between fruit types (ANOVA: F = 3866; df = 6; *p* < 0.001; Dunnett T3, 95% CI). These results align with manufacturer-published sensitivity profiles for BME688 and SGP30, which highlight their strong responsiveness to reduced organic vapors and aroma-active VOCs. The narrow inter-quartile ranges observed in these boxplots further indicate stable, consistent VOC measurements across replicate strawberry samples. This reliability underscores the suitability of these sensors for applications such as strawberry ripeness assessment, quality grading, and early spoilage detection, which similarly rely on subtle variations in VOC composition.

The Multichannel MEMS-based gas sensor (Figure 8b) exhibits mid-range VOC detection performance, with statistically significant differences between fruit types (ANOVA: F = 1975; df = 6; *p* < 0.001; Dunnett T3, 95% CI). However, its responses show greater variability than those of BME688 and SGP30. This wider dispersion likely reflects its multi-element design, through which each sub-sensor responds to different classes of VOCs. Such variation has been documented in earlier studies, where mechanical impact, storage temperature, and cultivar-specific volatiles produced heterogeneous VOC profiles in strawberries and other fruits. While this variation may reduce precision for ripeness scoring, it may be advantageous for broader chemical fingerprinting applications.

In contrast, SGP41 (Figure 8c) employs Sensirion’s advanced CMOSens® and MOXSens® technologies, which compute a combined VOC index ranging from 0–500. The observed readings near VOC Index ≈ 300 fall within an optimal detection zone, indicating strong sensitivity to strawberry-associated reducing VOCs and a robust response window well-suited for distinguishing high-aroma fruits from background levels. Statistical analysis supports this (ANOVA: F = 4869; df = 6; *p* < 0.001; Dunnett T3, 95% CI). Previous work evaluating CMOSens-based sensors similarly reports stable behavior and reduced noise when detecting mixed VOC environments, making them promising candidates for monitoring fruit freshness in dynamic, real-world conditions such as storage rooms or transport containers.

### 3.2. Multivariate Analysis of Sensor Responses Using Principal Component Analysis

While individual sensor responses provide valuable insight into sensitivity and selectivity, electronic nose systems fundamentally rely on multivariate pattern recognition to discriminate complex odor mixtures. To evaluate the collective behavior of the heterogeneous sensor array and to identify the sensing elements contributing most significantly to discrimination, principal component analysis (PCA) was performed using IBM SPSS Statistics version 30 on the baseline-subtracted dataset. Substance 0 (background air) was used only to establish the baseline and was excluded from the PCA model for the six fruits: strawberry (1), banana (2), orange (3), mango (4), apple (5), and pear (6).

**Figure A2:**
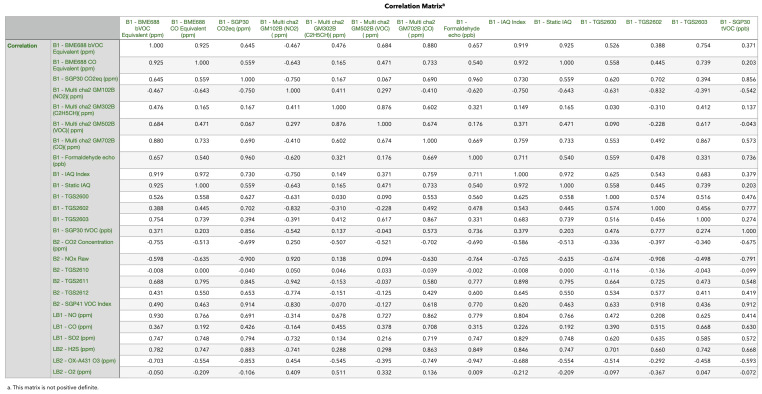

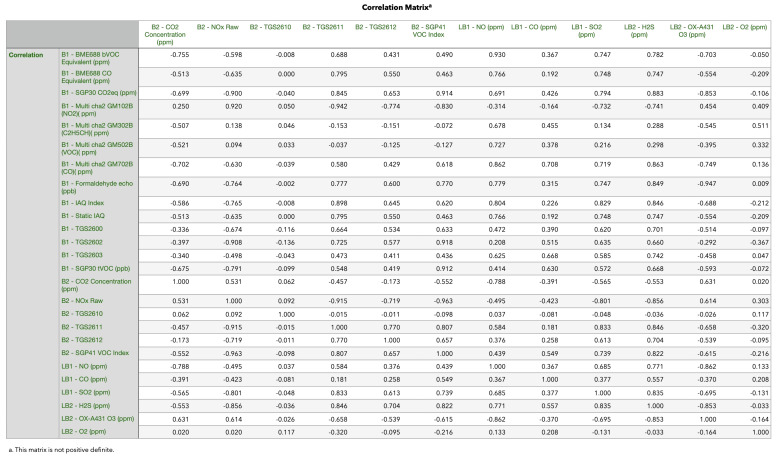
Correlation matrix of sensor variables.

Prior to principal component analysis, all sensor channels were standardized to zero mean and unit variance by performing PCA on the correlation matrix (See Appendix C, Figure A2). This scaling procedure ensured that differences in measurement units, dynamic ranges, and sensor technologies did not bias the multivariate analysis, allowing each sensor to contribute equally to component extraction.

#### 3.2.1. Sensor Contribution and Communality Analysis

The communalities table (Table 4) obtained from the SPSS PCA output presents the proportion of variance in each sensor variable, explained by the retained principal components. Sensors exhibiting high extracted communality values contribute strongly to the multivariate structure of the dataset and, consequently, to odor discrimination.

The highest communalities were observed for:

- B2—NOx Raw (0.972);
- B1—Multi channel GM102B (NO_2_) (0.950);
- B1—Multi channel GM502B (VOC) (0.949);
- LB1—NO (0.934);
- LB2—H_2_S (0.929);
- B1—Multi channel GM702B (CO) (0.925);
- B2—TGS2611 (0.916);
- B1—SGP41 VOC Index (0.872);
- B1—SGP30 CO_2_ equivalent (0.855).

These results demonstrate that both broadly responsive VOC sensors and more selective electrochemical gas sensors play a dominant role in shaping the PCA solution. The strong contribution of BME688, SGP30, and SGP41 confirms the importance of MOS-based VOC detection for odor intensity encoding, while the high communalities associated with NOx, NO, H_2_S, and NO_2_ sensors indicate that oxidizing- and nitrogen-based gas responses provide critical complementary information.

In contrast, B2—TGS2610 (communalities = 0.013) and LB2—O_2_ (0.310) exhibited minimal contribution, suggesting limited relevance for fruit aroma discrimination under the present experimental conditions. This finding provides valuable guidance for future sensor selection and array optimization, particularly for low-power or application-specific deployments.

#### 3.2.2. Eigenvalue Analysis and Component Retention

**Figure 9:**
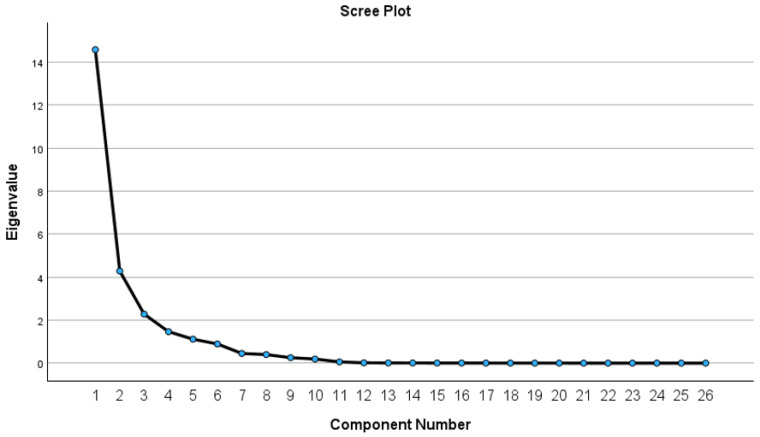
Scree plot showing eigenvalues obtained from PCA of baseline-subtracted e-nose data. The pronounced inflection after the second component supports the retention of two principal components.

To determine the appropriate number of principal components, eigenvalue analysis and inspection of the scree plot were performed, as shown in Figure 9. The scree plot reveals a pronounced drop in eigenvalue magnitude between the first and second components, followed by a gradual flattening beyond the second component. The first two principal components (PC1 and PC2) explained approximately 72.48% of the total variance in the dataset, with PC1 accounting for 56.01% and PC2 for 16.47%.

The first principal component (PC1) possesses a substantially larger eigenvalue, capturing the dominant variance in the dataset (56.01%), while the second principal component (PC2) retains an eigenvalue above unity and accounts for a meaningful proportion of the remaining variance (16.47%). Subsequent components exhibit eigenvalues close to zero and contribute only marginal additional information.

Based on the Kaiser criterion (eigenvalues > 1) and the clear inflection point observed in the scree plot, two principal components were retained. Together, PC1 and PC2 explain 72.48% of the total variance, indicating that the majority of the multivariate information contained in the full sensor array can be represented in a two-dimensional feature space.

#### 3.2.3. PCA Scores Plot and Fruit Discrimination

**Figure 10:**
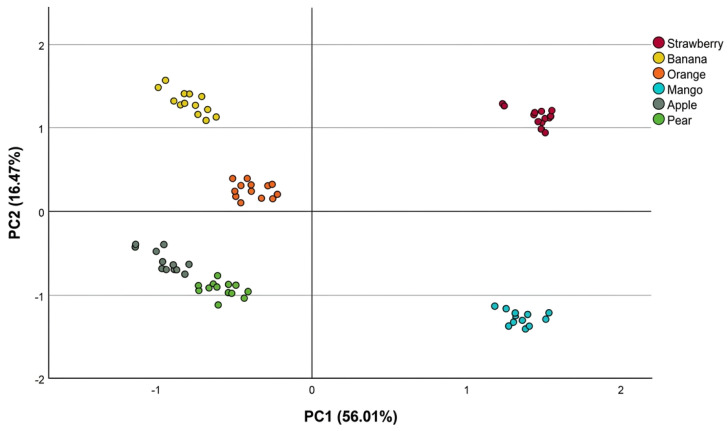
PCA scores plot of baseline-subtracted e-nose responses using regression factor scores, showing separation of fruit samples in the PC1–PC2 plane (PC1 = 56.01%, PC2 = 16.47%).

The regression-based PCA scores plot for PC1 (56.01%) and PC2 (16.47%) is shown in Figure 10. Each point represents an individual observation projected into the reduced PCA space, with samples grouped by fruit type: strawberry, banana, orange, mango, apple, and pear.

Clear and compact clustering is observed for all fruit samples, demonstrating that the electronic nose array captures distinct and reproducible odor fingerprints. Separation is dominated by PC1, which reflects overall VOC intensity and reducing gas activity. Fruits with richer and more complex volatile emissions, specifically strawberry and mango, occupy extreme regions along PC1, consistent with their known ester- and terpene-rich aroma profiles.

Banana and orange samples form intermediate clusters, reflecting moderate VOC emission dominated by characteristic esters and citrus terpenes, respectively. Apple and pear cluster closer together at lower PC1 values, indicating weaker overall VOC intensity and chemically similar volatile profiles at the measured ripeness stage.

PC2 provides secondary discrimination, separating fruits with comparable total VOC levels but differing gas composition. This axis is strongly influenced by oxidizing- and nitrogen-based sensor responses, as evidenced by the high communalities of NOx-related sensors. As a result, fruits with subtle differences in VOC composition and oxidation byproducts become separable in the PC1–PC2 plane, even when PC1 intensities overlap.

#### 3.2.4. Qualitative Sensor Contribution Analysis Based on Rotated Component Loadings

**Figure 11:**
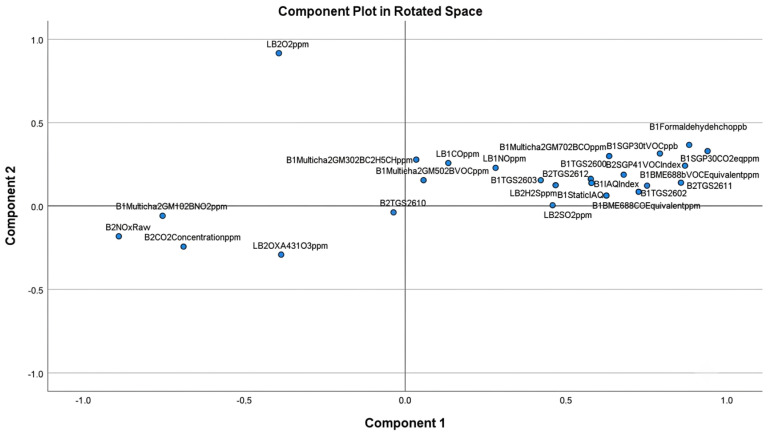
Rotated component loading plot (SPSS 30) showing sensor variables in PC1–PC2 space after Varimax rotation. VOC-sensitive sensors dominate PC1 (odor intensity), while oxidizing- and nitrogen-based sensors contribute to PC2 (compositional differences), illustrating the complementary roles of heterogeneous sensors in electronic nose discrimination.

The rotated component loading plot (Figure 11) shows how individual sensor variables contribute to the principal components and presents the structure of the multivariate responses. After Varimax rotation, distinct sensor groupings emerge in the PC1–PC2 space.

Sensors with strong positive loadings on PC1, including BME688 (bVOC, IAQ indices, and CO equivalent), SGP30 (CO_2_eq and tVOC), SGP41 (VOC index), and MOS sensors, such as TGS2600, TGS2602, and TGS2611, form a compact cluster. Their close proximity indicates high intercorrelation and confirms that PC1 represents overall VOC intensity and reducing gas activity, driving the primary discrimination between fruit samples.

In contrast, sensors such as Multichannel GM102BN (NO_x_ Raw), and OXA431 (O_3_) appear on the negative side of PC1, indicating an inverse relationship with VOC-dominated signals and representing background or oxidizing gas behavior.

The vertical separation along PC2 reflects compositional differences rather than intensity. VOC-related sensors (e.g., SGP30 tVOC and SFA30 formaldehyde) lie in the positive PC2 region, while NO_x_-related, SO_2_ and oxidizing sensors appear negative, indicating that PC2 captures the balance between reducing and oxidizing gases.

MEMS-based sensors (GM302B and GM502B) occupy intermediate regions, highlighting their role in detecting specific VOC classes, complementing the broader response of MOS sensors. Conversely, TGS2610 lies near the origin, confirming its negligible contribution to the PCA model.

Overall, the loading structure shows that odor discrimination arises from the combined behavior of sensor groups, with VOC-sensitive sensors dominating intensity (PC1) and selective sensors contributing to compositional differentiation (PC2), supporting the effectiveness of the heterogeneous adaptive e-nose design.

#### 3.2.5. Discussion of Integrated Multivariate Sensor Responses

Bringing together the ANOVA and PCA results provides a clearer picture of how the electronic nose system behaves as a whole rather than as isolated sensors. While the ANOVA analysis shows that individual sensors can distinguish between fruit samples, it does not fully reflect the complexity of odor detection, which is inherently a multivariate process.

The PCA analysis shows how the first two components capture most of the variation in the dataset and present well-defined clusters for the different fruits. Differences in overall VOC intensity are presented along PC1, with strawberry and mango showing stronger responses, while apple and pear show weaker emissions. The consistency between these trends and the ANOVA results gives confidence that the patterns observed are robust.

Looking at sensor behavior more closely, it becomes evident that not all sensors contribute equally. VOC-sensitive sensors, particularly BME688, SGP30, and SGP41, play a central role in defining the main patterns in the data, effectively capturing the intensity of the aroma. At the same time, selective sensors such as NO_x_, NO, H_2_S, and the MEMS channels provide additional detail by responding to differences in gas composition. The loading plots help to make this relationship clearer, showing how VOC-driven responses are separated from oxidizing gases such as NO_x_ and O_3_.

This also allows a simple but useful ranking of the sensors. VOC-focused sensors emerge as the most influential group, followed by selective sensors that help refine the discrimination between samples. Other sensors play a more limited role, and in some cases, such as TGS2610, contribute very little to the overall model.

#### 3.2.6. Implications for Adaptive e-Nose Design

The multivariate analysis confirms that effective odor discrimination emerges from the collective response of heterogeneous sensors, rather than reliance on any single sensing element. VOC-focused MOS sensors provide sensitivity to overall aroma intensity, while electrochemical sensors targeting NOx, NO, H_2_S, and CO contribute selectivity and compositional resolution.

Importantly, the PCA results validate the adaptive e-nose framework proposed in this study: newly integrated sensors can be quantitatively evaluated not only in isolation but also in terms of their multivariate contribution to odor classification. Sensors exhibiting consistently high communalities and strong influence on principal components represent optimal candidates for future deployments, while low-impact sensors may be excluded to reduce cost, power consumption, and system complexity.

Collectively, these results highlight the complementary strengths of the tested sensors:

- BME688 and SGP30: High sensitivity and excellent repeatability, ideal for quality grading, VOC profiling, and automated ripeness evaluation (future study).
- Multichannel MEMS sensor: Broader chemical responsiveness, valuable for complex mixture differentiation or identifying compositional shifts across cultivars.
- SGP41: Stable VOC index output with strong detection in the optimal range, enabling continuous monitoring and consumer-facing freshness systems.

The agreement between sensor trends and published GC–MS findings on strawberry VOC emissions underscores the promise of low-cost electronic nose (e-nose) technologies as scalable, real-time alternatives for agricultural quality assessment.

## 4. Conclusions

This study demonstrates that effective odor discrimination in low-cost electronic nose systems is achieved not through individual sensor performance alone, but through the coordinated behavior of a heterogeneous sensor array. By combining MOS, MEMS, electrochemical, and NDIR sensing technologies, the proposed system captures a broad representation of fruit-derived gaseous signatures as a proof of concept.

The analysis proves that VOC-sensitive sensors, such as BME688, SGP30, and SGP41, play a key role in detecting overall aroma intensity, while selective sensors, including NO_x_, NO, H_2_S, and multichannel MEMS elements, provide additional information related to gas composition and chemical balance. In contrast, certain sensors, such as TGS2610, contribute minimally under the tested conditions.

Importantly, these findings support the concept of an adaptive e-nose framework, where sensor relevance is determined based on multivariate contribution rather than isolated response.

This work proves that low-cost, modular sensor arrays can provide interpretable odor fingerprinting. Future work will focus on integrating advanced machine learning models and evaluating newly emerging sensors to further enhance system adaptability and enable deployment in real-world applications such as food quality monitoring, environmental sensing, and smart agriculture.

## Figures and Tables

**Table 1:** Sensor types.

Sensor Type	Operating Principle	Sensing Material
MOS Gas Sensor (Metal Oxide Semiconductor)	Chemiresistive—target gas adsorbs on heated SnO_2_ surface, causing a resistance change	Tin Dioxide (SnO_2_) on alumina substrate, noble-metal electrodes
MEMS MOX Gas Sensor (Multi-pixel)	Chemiresistive—gas reacts on MOX micro-hotplate; digital I^2^C output with onboard signal processing	Metal Oxide (MOX) on CMOS micro-hotplate with multiple independent sensing pixels
Electrochemical Gas Sensor (Amperometric)	Amperometric—target gas is oxidized at the working electrode, generating a current proportional to concentration; auxiliary electrode corrects zero drift	Platinum (Pt) and Carbon (C) catalyst electrodes in aqueous electrolyte with PTFE diffusion membrane
NDIR Gas Sensor	Infrared absorption at the CO_2_-specific wavelength	Infrared optical cavity (IR Source + detector pair)

**Table 2:** Sensors and sensing technologies.

Sensors	Sensing Technology
TGS2600, TGS2602, TGS2603, TGS2610, TGS2611, TGS2612	Metal Oxide Semiconductor (SnO_2_)
Air Quality Sensor (BME688)	
VOC and eCO_2_ Gas Sensor (SGP30) v1.0	Metal Oxide Semiconductor (MOS)
Multichannel Gas Sensor v2.0	Metal Oxide Semiconductor (MOS) with Micro-Electro-Mechanical System (MEMS)
Formaldehyde Sensor (SFA30) v1.0	Electrochemical
Barometer Sensor (BME680) v1.0	Metal Oxide Semiconductor (MOS) with MEMS-based for Temperature, Pressure & Humidity
VOC and NOx Gas Sensor (SGP41) v1.0	Metal Oxide Semiconductor (MOS) utilizing CMOSens^®^ and MOXSens^®^ technologies
CO_2_ Sensor (MH-Z16)	Non-Dispersive Infrared (NDIR)
Ammonia Gas Sensor (4-NH3-100)	Electrochemical principle
Nitric Dioxide Gas Sensor (NO2-A43F)	Electrochemical principle
Nitric Oxide Gas Sensor (NO-A4)	Electrochemical principle
Carbon Monoxide Gas Sensor (CO-A4)	Electrochemical principle
Methane and Combustible Gas Sensor (CH-A3)	Catalytic combustion (also known as pellistor technology)
Ozone Gas Sensor (OX-A431)	Electrochemical principle
Sulfur Dioxide Gas Sensor (SO2-A4)	Electrochemical principle
Oxygen Gas Sensor (LF02-A4)	Electrochemical principle
Hydrogen Sulfide Gas Sensor (4-H2S-100)	Electrochemical principle

**Table 3:** Fruit samples and their aroma/scents.

Fruit Type	Ripening Compound	Aroma Compound	Other Gases	Reference
Apple	Ethylene (ethene, C_2_H_4_)	VOCs (esters, alcohols, ketones, alkenes, aldehydes, acid, phenol)	Carbon Dioxide (CO_2_)	[32,33]
Banana	Ethylene (ethene, C_2_H_4_)	VOCs (esters, alcohols, ketones, alkenes, aldehydes, acid, phenol, hydrocarbons)	Carbon Dioxide (CO_2_)	[38]
Mango	Ethylene (ethene, C_2_H_4_)	VOCs (esters, alcohols, ketones, alkenes, aldehydes, acid)	Carbon Dioxide (CO_2_)	[34,39]
Orange	Ethylene (ethene, C_2_H_4_)	VOCs (esters, alcohols, ketones, alkenes, aldehydes, acid)	Carbon Dioxide (CO_2_)	[40]
Strawberry	Ethylene (ethene, C_2_H_4_)	VOCs (terpenes, esters, aldehydes, lactones, ketones, acids)	Carbon Dioxide (CO_2_)	[41,42]
Pears	Ethylene (ethene, C_2_H_4_)	VOCs (esters, aldehydes, alcohols, ketones, alkenes, acids)	Carbon Dioxide (CO_2_) and Nitrogen (N_2_)	[43]

**Table 4:** Communalities of sensor variables extracted from principal component analysis (PCA) performed in SPSS 30, indicating the proportion of variance in each sensor, explained by the retained components.

Communalities	Extraction	Communalities	Extraction	Communalities	Extraction
B1—BME688 bVOC Equivalent (ppm)	0.893	B1—IAQ Index	0.835	B2—TGS2611	0.916
B1—BME688 CO Equivalent (ppm)	0.695	B1—Static IAQ	0.695	B2—TGS2612	0.599
B1—SGP30 CO2eq (ppm)	0.855	B1—TGS2600	0.532	B2—SGP 41 VOC Index	0.872
B1—Multi cha2 GM102B (NO2) (ppm)	0.950	B1—TGS2602	0.796	LB1—NO (ppm)	0.934
B1—Multi cha2 GM302B (C2H5CH) (ppm)	0.894	B1—TGS2603	0.611	LB1—CO (ppm)	0.314
B1—Multi cha2 GM502B (VOC) (ppm)	0.949	B1—SGP30 tVOC (ppb)	0.537	LB2—SO2 (ppm)	0.790
B1—Multi cha2 GM702B (CO) (ppm)	0.925	B2—CO2 Concentration (ppm)	0.583	LB2—H2S (ppm)	0.929
B1—Formaldehyde hcho (ppb)	0.733	B2—NOx Raw	0.972	LB2—OX-A431 O3 (ppm)	0.728
		B2—TGS2610	0.013	LB2—O2 (ppm)	0.310

Extraction method: Principal component analysis.

**Figure A1:**
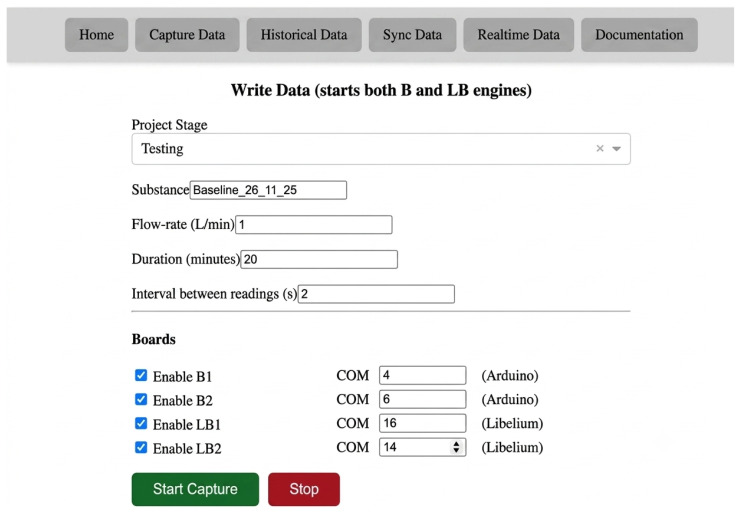
e-nose—Dashboard.

## Data Availability

The data presented in this study are available on request from the corresponding author.

## Appendix A. Sensor Array Specifications and Descriptions

This appendix provides the full hardware specifications and datasheet-level descriptions of the sensors integrated in the adaptive e-nose, organized by acquisition board. Table A1 and Table A2 list the sensors connected to the two Seeeduino Cortex-M0+ boards, while Table A3 and Table A4 list the electrochemical sensors mounted on the two Waspmote Gases PRO boards.

**Table A1:** Sensors connected to Seeeduino Cortex-M0+ Board 1 (adapted from [51,52]).

Sensor Module—Make	Sensing Technology	Target Gases	Measurement Range	Cross Sensitivity
TGS2600-B00—Figaro Engineering Inc.	Metal Oxide Semiconductor (SnO_2_)	Air contaminants (H_2_, CO, ethanol, methane)	1–30 ppm H_2_	reducing gases, VOCs
TGS2602-B00—Figaro Engineering Inc.	Metal Oxide Semiconductor (SnO_2_)	Odorous Gases (NH_3_, H_2_S, etc.), VOCs	1–30 ppm ethanol	n/a
TGS2603—Figaro Engineering Inc.	Metal Oxide Semiconductor (SnO_2_)	Air Contaminants (Trimethylamine, methyl mercaptan, H_2_S, ethanol, etc.)	1–10 ppm H_2_	n/a
Grove—Air Quality Sensor (BME688)—Seeed Studio with a chip from Bosch Sensortec	Metal Oxide Semiconductor (MOS) with Artificial Intelligence (AI)	Volatile Organic Compounds (VOCs), Volatile Sulfur Compounds (VSCs), Carbon Monoxide (CO), and Hydrogen (H_2_)	Parts per billion (ppb) range for target gases	n/a
Grove VOC and eCO_2_ Gas Sensor (SGP30) v1.0	Seeed Studio with a chip from Sensirion—Metal Oxide Semiconductor (MOS)	TVOCs, eCO_2_	TVOC: 0–60,000 ppb & eCO_2_: 400–60,000 ppm	n/a
Multichannel Gas Sensor v2.0—Seeed Studio with chips from Winsen	Metal Oxide Semiconductor (MOS) with Micro-Electro-Mechanical System (MEMS)	Nitrogen dioxide (NO_2_), Ethyl Alcohol (C_2_H_5_OH), VOC, CO	NO_2_: 0.1–10 ppm & C_2_H_5_OH: 1–500 ppm & VOC: 1–500 ppm & CO: 5–5000 ppm	n/a
Grove—Formaldehyde Sensor (SFA30) v1.0—Seeed Studio with a chip from Sensirion	Electrochemical	Formaldehyde (HCHO) with integrated RTH (Relative Humidity & Temperature)	0–1000 ppb	n/a

**Table A2:** Sensors connected to Seeeduino Cortex-M0+ Board 2 (adapted from [51,52]).

Sensor Module/Make	Sensing Technology	Target Gases	Measurement Range	Cross Sensitivity
TGS2610—Figaro Engineering Inc.	Metal Oxide Semiconductor (SnO_2_)	LPG (Liquefied Petroleum Gas)—mixture of Propane (C_3_H_8_) and Butane (C_4_H_10_)	500–10,000 ppm (or 1–25% Lower Explosive Limit (LEL))	Ethylene, Alcohols, organic vapours
TGS2611—Figaro Engineering Inc.	Metal Oxide Semiconductor (SnO_2_)	Methane (CH_4_) and natural gas	500–10,000 ppm (or 1–25% Lower Explosive Limit (LEL))	low sensitivity to Hydrogen (H_2_), Iso-butane (i-C_4_H_10_), Ethanol (C_2_H_5_OH)
TGS2612—Figaro Engineering Inc.	Metal Oxide Semiconductor (SnO_2_)	Methane (CH_4_), Propane (C_3_H_8_), Iso-Butane (i-C_4_H_10_) (components of LNG/LPG)	1–25% Lower Explosive Limit (LEL) of each gas	Hydrogen (H_2_), Ethanol (C_2_H_5_OH)
Grove—Barometer Sensor (BME680) v1.0—Seeed Studio with a chip from Bosch Sensortec	Metal Oxide Semiconductor (MOS) with MEMS-based for Temperature, Pressure & Humidity	Volatile Organic Compounds (VOCs), temperature, humidity, and air pressure	Temp.: −40–+85 °C, Humidity: 0–100% r.H, Pressure: 300–1100 hPa, VOC: Broad range, typically measured in kΩ	Hydrogen (H_2_)—moderate, Carbon monoxide—limited
Grove—VOC and NOx Gas Sensor (SGP41) v1.0—Seeed Studio with a chip from Sensirion	Metal Oxide Semiconductor (MOS) utilizing CMOSens® and MOXSens® technologies	VOC Pixel (Reducing Gases): VOCs, NOx Pixel (Oxidizing Gases): Nitrogen Dioxides (NO_2_)	VOCs: 0–1,000,000 ppb (Ethanol in clean air), VOC index: 0–500, NOx: 0–10,000 ppb (NO_2_ in clean air), NOx index: 0–500	Hydrogen (H_2_), Ozone (O_3_)
Grove—CO_2_ Sensor (MH-Z16)—Seeed Studio	Non-Dispersive Infrared (NDIR)	Carbon Dioxide (CO_2_)	0–2000 ppm	Highly selective for CO_2_; negligible cross-sensitivity to other gases

**Table A3:** Sensors mounted on Waspmote Gases PRO Sensor Board-1 (adapted from [53]).

Sensor Module/Make	Sensing Technology	Target Gases	Measurement Range	Cross Sensitivity
Ammonia Gas Sensor (4-NH3-100)—Honeywell Analytics, Lincolnshire, IL, USA	Electrochemical principle	Ammonia (NH_3_)	0–100 ppm	0 ppm@300 ppm CO, 1.5 ppm@5 ppm H_2_S,−3 ppm@5 ppm CO_2_,30 ppm@15 ppm H_2_,−1 ppm@35 ppm Isobutylene,0@100 ppm Ethanol
Nitric Dioxide Gas Sensor (NO2-A43F)—Alphasense Ltd., Braintree, UK	Electrochemical principle	Nitrogen Dioxide (NO_2_)	0–20 ppm	<−80 ppm@5 ppm H_2_S,<5 ppm@5 ppm NO,<75 ppm@5 ppm Cl_2_,<−5 ppm@5 ppm SO_2_,<−5 ppm@25 ppm CO,<−0.1 ppm@100 ppm H_2_,<1 ppm@100 ppm C_2_H_4_,<0.2 ppm@20 ppm NH_3_, <0.1 ppm@5% vol CO_2_,nd @ 100 ppm Halothane
Nitric Oxide Gas Sensor (NO-A4)—Alphasense Ltd., Braintree, UK	Electrochemical principle	Nitric Oxide (NO)	0–18 ppm	<0 ppm@300 ppm CO,<0 ppm@5 ppm SO_2_,<1.5 ppm@5 ppm NO_2_,<−1.5 ppm@15 ppm H_2_
Carbon Monoxide Gas Sensor (CO-A4)—Alphasense Ltd., Braintree, UK	Electrochemical principle	Carbon Monoxide (CO)	0–25 ppm	<0.1 ppm@5 ppm H_2_S,<−2 ppm@5 ppm NO_2_,<0.1 ppm@5 ppm Cl_2_,<−2 ppm@5 ppm NO,<0.1 ppm@5 ppm SO_2_,<10 ppm@100 ppm H_2_,<0.5 ppm@100 ppm C_2_H_4_,<0.1 ppm@20 ppm NH_3_

**Table A4:** Sensors mounted on Waspmote Gases PRO Sensor Board-2 (adapted from [53]).

Sensor Module/Make	Sensing Technology	Target Gases	Measurement Range	Cross Sensitivity
Methane and Combustible Gas Sensor (CH-A3)—Alphasense Ltd., Braintree, UK	Catalytic combustion (also known as pellistor technology)	Methane (CH_4_)	0–100% Lower Explosive Limit (LEL) Methane	160–175%LEL@130–140% Sensitivity Hydrogen, 350–450%LEL@150–190% Sensitivity Propane, 420–500%LEL@150–180% Sensitivity Butane, 600–670%LEL@180–200% Sensitivity n-Pentane, 800–950%LEL@150–170% Sensitivity Nonane, 17–18%LEL@42–44% Sensitivity Carbon Monoxide, 300–340%LEL@150–170% Sensitivity Acetylene, 270–320%LEL@150–170% Sensitivity Ethylene, 450–500%LEL@180–200% Sensitivity Isobutylene
Ozone Gas Sensor (OX-A431)—Alphasense Ltd., Braintree, UK	Electrochemical principle	Ozone (O_3_)	0–18 ppm	<100 ppm@5 ppm H_2_S, <70–120 ppm@5 ppm NO_2_, <30 ppm@5 ppm Cl_2_, <3 ppm@5 ppm NO, <−6 ppm@5 ppm SO_2_,<0.1 ppm@5 ppm CO,
Ozone Gas Sensor (OX-A431)—Alphasense Ltd., Braintree, UK	Electrochemical principle	Ozone (O_3_)	0–18 ppm	<0.1 ppm@100 ppm H_2_, <0.1 ppm@100 ppm C_2_H_4_, <0.1 ppm@20 ppm NH_3_, <0.1 ppm@50,000 ppm CO_2_, <0.1 ppm@100Halothane
Sulfur Dioxide Gas Sensor (SO2-A4)—Alphasense Ltd., Braintree, UK	Electrochemical principle	Sulfur Dioxide (SO_2_)	0–20 ppm	<40 ppm@5 ppm H_2_S, <−160 ppm@5 ppm NO, <−70 ppm@5 ppm Cl_2_, <−1.5 ppm@5 ppm SO_2_, <2 ppm@5 ppm CO, <1 ppm@100 ppm H_2_, <1 ppm@100 ppm C_2_H_4_, <0.1 ppm@20 ppm NH_3_, <0.1 ppm@5% vol CO_2_
LF02-A4—Alphasense Ltd., Braintree, UK	Electrochemical principle	Oxygen (O_2_)	0–30% Vol.%	n/a
Hydrogen Sulfide Gas Sensor (4-H2S-100)—Honeywell Analytics, Lincolnshire, IL USA	Electrochemical principle	Hydrogen Sulfide (H_2_S)	0–100 ppm	≤6 ppm@50 ppm CO, 1 ppm@5 ppm H_2_S, 1 ppm@35 ppm NO, −1 ppm@5 ppm NO_2_, 25 ppm@10,000 ppm H_2_, <0 ppm@100 ppm C_2_H_4_, ±1.5 ppm@5000 ppm C_2_H_6_O

### Appendix A.1. Environmental and Integrated Gas Sensing (BME Series)

Electronic nose systems use a digital fingerprint of a scent, mimicking the biological olfactory system of animals. However, just as animal noses struggle to function/detect scents properly in extreme environmental conditions, such as very low temperature or very dry air [54], electronic nose systems are also profoundly subject to their surrounding environment [55]. Therefore, the first process to reduce these issues is to have a comprehensive selection of sensors to compensate for environmental changes.

The BME series is a 4-in-1 environmental sensor developed by Bosch Sensortec, and its integration into the Seeed Studio’s Grove ecosystem provides convenient measurement of temperature, humidity, pressure, and gas (VOCs). In addition, they provide baseline environmental data that are crucial for calibrating the other gas sensors. In this experimental setup, BME680 is connected to Seeeduino Cortex-M0+ Board 2 (Table A2) and BME688 to Cortex-M0+ Board 1 (Table A1) through an I^2^C interface so that the baseline readings can be best correlated.

### Appendix A.2. Digital MOX Gas Sensors

(a) Sensirion SGP Series
  The multi-pixel metal oxide (MOx) sensors from Sensirion, theSGP30 and SGP41 modules, detect total volatile organic compounds (tVOC) and CO_2_ equivalent (CO_2_eq) and are designed for easy integration into air quality monitoring systems. A digital I^2^C interface is used for communication with Cortex-M0+ Board 1 (Table A1) & Cortex-M0+ Board 2 (Table A2), respectively.
  While SGP30 measures tVOC in ppb and CO_2_eq in ppm, SGP41 detects both VOCs and nitrogen oxide compounds (NOx). Rather than providing tVOC & CO_2_ eq values directly, SGP41 processes raw sensor signals using a powerful gas index algorithm to provide a general “air quality” value, allowing for automatic triggering of an air treatment device. Hence, the algorithm implemented in Board 2 is slightly different from that in Board 1.
(b) Grove Multichannel Gas Sensor V2
  Despite major developments in electronic nose systems and the easy availability of Grove sensors in the market, we could not find many Multichannel Gas Sensor applications, as their usage has not been explored much. In this project, we used this approach to understand its characteristics and pave the way for future applications. It is a compact, I^2^C-based sensor module that uses four independent MEMS Sensor elements (GM-102B, GM-302B, GM-502B, and GM-702B) to qualitatively detect the presence of various gases, allowing simultaneous monitoring of different gases. They are sensitive to specific gas types: GM-102B detects NO_2_, GM-302B detects C_2_H_5_OH, GM-502B detects VOCs and GM-702B detects CO. All their communications are handled via an I^2^C bus with a default address of 0x55. In the experimental setup, it is connected to Seeeduino Cortex-M0+ Board 1 (Table A1). However, to achieve internal chemical balance and get stable data, a minimum of 24 h of warm-up is recommended for a new sensor. The module should avoid exposure to high concentrations of corrosive gases (H_2_S, SOX, Cl_2_, HCl) and volatile silicon compounds, as this can cause damage or reduce sensitivity.

### Appendix A.3. Analog/Traditional Metal Oxide Sensors (TGS Series)

The application of TGS sensors is diverse, but they are primarily used in safety, environmental monitoring, and air quality control due to low cost, long life, and simple circuitry requirements. We have used an array of Figaro sensors (TGS2600, TGS2602, and TGS2603 in Seeeduino Cortex-M0+ Board 1 (Table A1), TGS2610, TGS2611, and TGS2612 in Seeeduino Cortex-M0+ Board 2 (Table A2)) to detect different odors, as every sensor provides distinct gas signatures. TGS2600 detects air contaminants, VOCs are targeted by TGS2602, TGS2603 detects H_2_S, LPGs are detected by TGS2610/TGS2612 and CH_4_ by TGS2611. With a host of sensors detecting various compounds, our system became more versatile in diversifying its application area.

### Appendix A.4. Digital Electrochemical Sensor—Grove Formaldehyde (HCHO) Gas Sensor

Seeed Studio manufactured the Grove Formaldehyde Sensor using a sensing element from another manufacturer, with the latest model utilizing the Sensirion SFA30 sensor module. However, there are not many studies that have been undertaken to understand the true potential of this emerging sensor, despite the older formaldehyde sensor having a Winsen WSP2110 sensor chip [56], used to test formalin in food, and the MQ-138 sensor is more sensitive than it. The systematic investigation of these low-cost electrochemical formaldehyde sensors (SFA30) demonstrated that formaldehyde concentration had a greater influence on sensor response than temperature and relative humidity [57]. This reinforces the need for sensors with low cross-sensitivity to temperature and relative humidity.

It has a measurement range of 0–1000 ppb, with a typical accuracy of ±20 ppb or ±20% of the measured value. Its built-in RHT (relative humidity and temperature) compensates the environmental changes, and its serial communication protocols—I^2^C (Inter-Integrated Circuit)—and UART (Universal Asynchronous Receiver/Transmitter) interfaces allow flexible connectivity and are currently connected to Seeeduino Cortex-M0+ Board 1 (Table A1).

### Appendix A.5. Grove CO_2_ Sensor

We added a Grove CO_2_ Sensor in our system to make it more versatile and correlate the detection performance of other sensors. It uses the non-dispersive infrared radiation (NDIR) principle and has a measurement range of 0–2000 ppm, with a built-in temperature sensor for compensation; it uses a UART (Universal Asynchronous Receiver/Transmitter) interface and is connected to Seeeduino Cortex-M0+ Board 2 (Table A2). This grove prototyping system from Seeed Studio provides standardized connectors for easy, solder-less integration with micro controllers over an Arduino base shield.

### Appendix A.6. Libelium Sensors

Libelium offers a wide range of sensors that are used in Internet of Things (IoT) applications. The sensors are factory-calibrated and provide maximum accuracy for professional applications; their modular design allows users to combine a vast range of sensors on a single device for multi-purpose projects across different environments. However, we used sensors for Carbon Monoxide (CO), Nitric Oxide (NO), Nitric Dioxide (NO_2_), and Ammonia (NH_3_) in Libelium Board 1 (Table A3) and Ozone (O_3_), Sulfur Dioxide (SO_2_), Methane (CH_4_), and Hydrogen Sulphide (H_2_S) in Libelium Board 2 (Table A4) so that we could best understand which sensors provide better response to the odors exuded by the substances.

## Appendix B. Software Dashboard, Cloud Storage, and Real-Time Analytics

### Appendix B.1. Dashboard with Real-Time Monitoring Interface

The main user interface of the system is a browser-based dashboard (Figure A1) created using Plotly Dash. This dashboard acts as the control center for the entire experiment, allowing users to start and stop data recording, define experimental parameters, and observe sensor behavior in real time. The dashboard was designed to be simple to use so that experiments can be conducted without interacting directly with the Python scripts. All interactions, such as selecting substances, defining experiment stages, and viewing live plots, are handled through the graphical interface.

The real-time monitoring page displays live sensor data using interactive line graphs and tables. Each time new data arrives in the Firebase database, the dashboard automatically updates the plots without requiring the page to be refreshed. Users can compare current readings against baseline sessions, allowing immediate visual assessment of gas exposure events and sensor recovery behavior. This feature is particularly useful during long experiments, as it provides instant feedback on whether the system is operating correctly.

### Appendix B.2. Data Collection and Synchronization Strategy

The e-nose platform uses a hybrid data synchronization strategy because the gas-sensing boards and environmental boards operate at different speeds. The gas boards (B1 and B2) continuously stream data at a steady rate. Their readings are combined into a single data row with a shared timestamp so that gas sensor values from both boards remain perfectly aligned. In contrast, the environmental boards (LB1 and LB2) produce data more slowly and at irregular intervals. For this reason, their data is recorded independently whenever a complete frame is received. This design ensures that slower environmental readings do not delay the high-frequency gas measurements. This strategy maintains high-resolution gas data while still capturing important environmental context such as temperature, humidity, and air quality.

### Appendix B.3. Firebase Realtime Database Integration

Firebase Realtime Database is used as the cloud backend for storing and streaming sensor data. Each experiment is saved using a structured path based on the experiment stage, substance, test ID, and board name. As soon as new readings are generated, they are uploaded to Firebase using the Firebase Admin SDK. The dashboard listens to these database updates and refreshes the visualizations instantly. This makes it possible to monitor experiments remotely from any web-enabled device. Using Firebase removes the need for custom server development and provides a reliable, scalable solution for handling real-time data in IoT-based research systems.

### Appendix B.4. Real-Time Graphing and Visual Analytics

Sensor readings are visualized using interactive graphs generated with Plotly. These graphs show how sensor values change over time during baseline, exposure, and recovery phases. The system supports overlaying baseline curves on exposure data, allowing easy comparison between different experimental stages. This visual feedback helps researchers identify sensor drift, response delays, and recovery behavior. The real-time analytics component plays a key role in experiment quality control by providing immediate insight into sensor performance and environmental stability.
